# Maternal mortality in Ifakara Health and Demographic Surveillance System: Spatial patterns, trends and risk factors, 2006 – 2010

**DOI:** 10.1371/journal.pone.0205370

**Published:** 2018-10-22

**Authors:** Alfred Kwesi Manyeh, Rose Nathan, Gill Nelson

**Affiliations:** 1 School of Public Health, Faculty of Health Sciences, University of the Witwatersrand, Parktown, Johannesburg, South Africa; 2 Dodowa Health Research Centre, Dodowa, Ghana; 3 Ifakara Health and Demographic Surveillance System site, Ifakara, Tanzania; The University of Warwick, UNITED KINGDOM

## Abstract

**Introduction:**

Maternal mortality was the subject of the United Nations’ fifth Millennium Development Goal which was to reduce the maternal mortality ratio by three quarters from 1990 to 2015. The Sustainable Development Goals (SDGs), target 3.1 requires participating countries to reduce their maternal mortality ratio to less than 70 deaths per 100,000 live births by 2030. Although much research has been conducted, knowing the spatial patterns and risk factors associated with maternal mortality in developing countries helps target scarce resources and intervention programmes to high risk areas for the greatest impact.

**Methods:**

Data were analysed from a longitudinal open cohort of women aged 15 to 49 years, enrolled from 2006 to 2010. An inverse distance weighted method of interpolation was used to assess spatial patterns of maternal mortality. Cox proportional hazards regression analysis was used to identify risk factors associated with maternal mortality.

**Results:**

The overall maternal mortality rate for the 36 792 study participants for the five years was 0.79 per 1000 person years. The trend declined from 90.42 in 2006 to 57.42 in 2010. Marked geographical differences were observed in maternal mortality patterns. The main causes of maternal death were eclampsia (23%), haemorrhage (22%) and abortion-related complications (10%). There was a reduced risk of 82% (HR = 0.18, 95% CI:0.05–0.74) and 78% (HR = 0.22, 95% CI:0.05–0.92) for women aged 20–29 and 30–39 years, respectively, compared with those younger than 20 years. While being married had a protective effect of 94% (HR = 0.06, 95% CI: 0.01–0.51) compared with being single, women who were widowed had an increased risk of maternal death of 913% (HR = 9.13, 95% CI: 1.02–81.94). Women who belong to poorer, poor and least poor socioeconomic quintile had 84%, 71% and 72% reduction in risk of maternal mortality respectively compared to those in the poorest category (HR = 0.16, 95% CI: 0.06–0.42; HR = 0.29, 95% CI: 0.12–0.69; HR = 0.28, 95% CI: 0.10–0.80).

**Conclusion:**

Maternal mortality has declined in rural southern Tanzania since 2006, with geographical differences in patterns of death. Eclampsia, haemorrhage and abortion-related complications are the three leading causes of maternal death in the region, with risk factors being younger than 20 years, being single or widowed, and having a low socioeconomic status.

## Introduction

Estimates developed by the World Health Organization (WHO) in 2007 show that, every day, 1500 women die from pregnancy related complications [1]. There were 536 000 estimated maternal deaths worldwide in 2005 [1]. Most of these avoidable deaths occurred in developing countries. Of all maternal deaths, 99% occur in developing countries where 85% of the world’s population lives; more than half of these occur in sub-Saharan Africa and one third in South Asia [1]. The MMR in developing countries is 450 per 100 000 live births versus 9 per 100 000 live births in developed countries [1].

Improving maternal health was one of the eight Millennium Development Goals adopted by the international community at the United Nations Millennium Summit in 2000 [2]. Millennium Development Goal 5 (MDG5) is to improve maternal health and participating countries made a commitment to reduce the maternal mortality ratio (MMR) by three quarters from 1990 to 2015 [3]. However, from 1990 to 2008, the global MMR declined by only 2.3%, indicating that achieving MDG5 required accelerating progress [2]. A systematic analysis in 2014 suggested that only 16 countries would achieve the MDG5 [4]. Due to the unavailability of reliable data, evaluating the progress towards this target has been a challenge to many developing countries where maternal mortality is still high.

Tanzania just like most African countries was unable to achieve her millennium development goal (MDGs) five in 2015. The maternal mortality ratio (MMR) for Tanzania is 556 deaths per 100,000 live births. The confidence interval for the 2015–16 Tanzania Demographic and Health Survey and Malaria Indicator Survey (TDHS-MIS) ranges from 446 to 666 deaths per 100,000 live births [5].

Target 3.1 of the 17 new sustainable development goals (SDGs) introduced in 2015 to replace the MDGs requires participating countries to reduce their maternal mortality ratio to less than 70 deaths per 100,000 live births by 2030 [6]. Over the years, studies have shown that there are differences in maternal mortality between countries, and large disparities within countries and people with high and low incomes, and between rural and urban populaces [7]. Women die from a wide range of pregnancy and childbirth related complications. It has been shown that the major causes of maternal mortality are abortion, haemorrhage and “other direct causes” [4].

Maternal mortality is associated with a number of risk factors, including age [8–11], parity [8–10], education of mothers [9, 12], obstetric factors [8, 11], unavailability of health facilities and trained health personnel [9, 10, 13], socio-economic factors [9, 12], and ethnic and religious affiliations [11].

There is limited information on levels of maternal mortality and causes of maternal deaths in most developing countries due to the lack of adequate vital registration systems and poor certification of causes of deaths. Most deaths occur at home, making it difficult to obtain satisfactory information [14]. Likewise, being a rare event, factors associated with maternal mortality are difficult to measure since large sample sizes are needed; there is thus a paucity of maternal mortality data in sub-Saharan Africa, particularly at sub-national level.

The existence of Health and Demographic Surveillance Systems (HDSSs) in African countries, such as Tanzania, provide a unique opportunity to calculate rates and trends, and to identify risk factors associated with maternal mortality, as demonstrated in a few studies already conducted in South Africa [15], Senegal[12] and Ethiopia[16].

Verbal autopsies (VAs) are used to collect information on cause of death. As demonstrated by other studies in Tanzania [17] and elsewhere [14, 18], the VA method of identifying probable cause of death is well recognized and the tools have been validated in various settings. The method is useful for evaluating trends of disease and mortality as shown in a number of previous studies [18–20].

Studies on spatial analysis and mapping of diseases are becoming increasingly important in public health awareness about disease burdens and risks to the general population [18, 21].

This study made use of HDSS data including causes of death established through verbal autopsy as well as Geographical Information System data to assess the spatial patterns, trends, causes and risk factors associated with maternal mortality in rural southern Tanzania over time, with a view to providing information that may help reduce the MMR in Tanzania.

## Materials and methods

### Study setting

**Fig 1:**
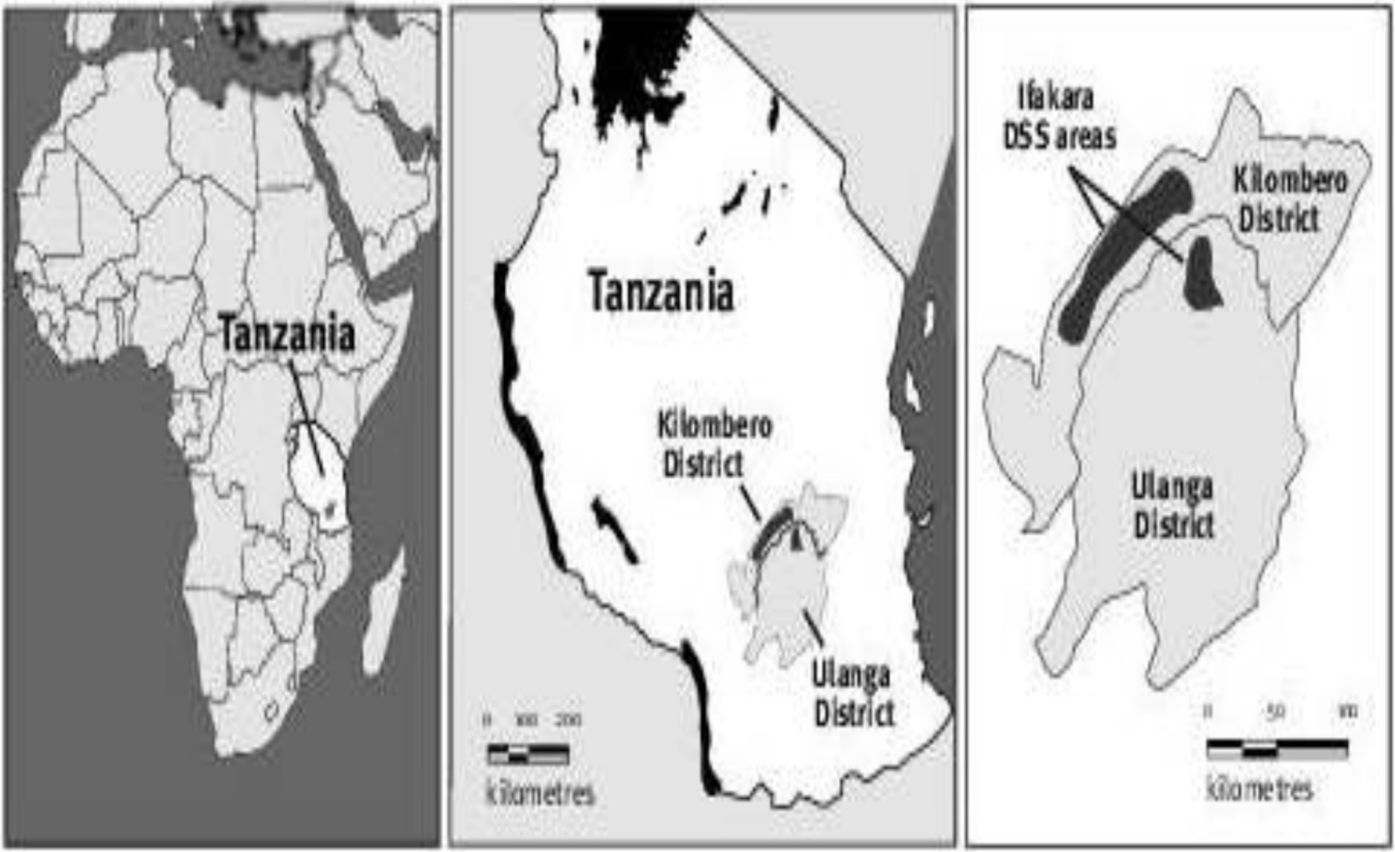
Map showing location of the IHDSS within the Morogoro Region, Tanzania.

The Ifakara Health and Demographic Surveillance System (IHDSS) was established in September 1996[22]. It is located in rural southern Tanzania, in two districts, Kilombero and Ulanga, both of which are in the Morogoro region (Fig 1) [23]. The IHDSS covers areas of 80 km ×18 km in Kilombero District and 40 km ×25 km in Ulanga District, a total of 2400 km^2^ of Guinea Savannah in the floodplain of the Kilombero River which divides the two districts [22, 23]. The area is predominately rural with scattered households. All health facilities and households were geo referenced in 2006 and annually thereafter [24].

The health system in the Demographic Surveillance Area (DSA) is made up of village health workers, 13 dispensaries, 2 health centres and hospitals that provide preventive and curative health services to the population [22].

### Study population

The study population comprised women of reproductive age (15–49 years). Women included in the study were those who were active members (residents) in registered households between 1 January 2006 and 31 December 2010. All women (15–49), who were not resident members of the IHDSS between 1 January 2006 and 31 December 2010 were excluded. A total of 36 792 women who met the inclusion criteria were part of the study.

### Ethic consideration

Upon the household visit in every update round of surveillance data collection, the researchers of IHDSS briefed each respondent as well as their household heads about the IHDSS data collection. Each participating individual was encouraged to ask for any further clarification, after which a verbal consent was sought prior to the interview; those who refused were excluded from the surveillance. Within each round of surveillance, samples of households were randomly picked for revisit by field supervisors to verify the information as well as whether verbal consent was sought by the interviewer. This procedure of obtaining informed consent in the IHDSS was approved by the Ifakara Institutional Review Board.

The study (secondary data analysis) was approved by the Human Research Ethics Committee (Medical) of the University of the Witwatersrand, Johannesburg, South Africa (Clearance Certificate Nunber. M111130) and the Ifakara Institutional Review Board (Clearance Certificate Number: IHI/IRBNo:11–2012). Permission to use the data was granted by the management of Ifakara Health and Demographic Surveillance System in Tanzania. Copies of the clearance certificates and the approval letter are attached in the appendix of this document.

### Data source and extraction

The IHDSS database is built on a platform of FoxPro (Microsoft Cor., Seattle, USA) database environment but the data are captured and processed using a database called Household Registration System (HRS2) which is a relational model commonly used for HDSS. The tables are in DBF format and the database is composed of a number of different tables that are related to each other by unique identifiers (ID).

The DBF tables with variables necessary to answer the research questions were extracted and Stat/transfer software was used to transfer the data to statistical software, STATA version 11. The extracted data were cleaned to identify all internal consistency of the responses and missing values. All inconsistencies in the data were amended by using the hard copies of the completed data collections tools and effecting the necessary changes. Variables were recoded where necessary, using STATA 11.

All the variables for this study were extracted from the following tables in the IHDSS database:

Membership: This applies to membership of a social unit (household). It contains records of every individual who has ever resided in the DSA. The records are uniquely identified by an individual’s permanent ID. The table contains individual variables such as names, date of birth, gender, marital status, occupation, entry and exit dates.

Family: Contains records of each family/household in the DSA. It contains variables such as family ID, household head’s name, date of last visit, village, etc.

Pregnancy Outcome: Stores information about pregnancy outcome, including mother’s ID, antenatal clinic visit, parity, date and type of outcome, date of birth, place of birth, etc.

Socio Economic Status: Contains information about household characteristics and assets which are updated annually and are used in the estimation of the household wealth index, including roof type, source of power (firewood, kerosene/biogas or electricity), and ownership of a bicycle, radio, car, motorbike, mobile phone and livestock.

Education: Stores information about the number of years of education completed by an individual in the DSA, the level of completed, etc.

GIS: The IHDSS had all housing structures, roads, health facilities and schools in the DSA geo referenced.

Verbal Autopsy: This table stores information on symptoms of the disease that led to death, date of death, place of death, individual ID, etc.

All these tables were linked by either the individual permanent ID or the household ID and, through these unique identifiers, all the required variables were extracted and stored into one table. The coding and editing was done on the extracted variables using STATA version 11.

### Measurements and statistical methods

The outcome variable was maternal death. This was coded as a binary variable, with all women who experienced a death coded as 1, and those who survived or out migrated coded as 0. The exposure variables were divided into maternal socio-economic and demographic variables. Socio-economic variables included socio-economic status (SES) which was measured using an index based on social status, assets ownership, and availability of utilities [25, 26]. The index measures were combined into a wealth index, using weights derived through principal component analysis (PCA) [27]. The proxies from the PCA were divided into five quintiles: poorest, very poor, poor, less poor and least poor. Socio-demographic variables included age (<20, 20–29, 30–39, 40–49), level of education (No education, Primary, Secondary and above), occupation (Business, farming, Trading, Others), marital status (Single, Married, Divorced/Separated, Widowed), place of delivery (Health facility, Outside health facility), Assistant at delivery (Health worker, Non health worker), distance to health facility (Near (<5Km), Far (> = 5Km)), antenatal clinic attendance (0 to 1, 2 to 3, 4 and above), and parity (Parity 1, Parity 2–4, Parity 5+).

VA was conducted on every death to determine the probable cause. The 10^th^ revision of the 1992 International Classification of Diseases (ICD-10) [28] was used to classify probable causes of death, including maternal deaths. The VA is the process of obtaining information about the cause of a death from close relatives of the recently deceased person in situations where medical report on cause of death is not available [3, 29, 30]. This is a reliable and accepted method of establishing cause-specific mortality in resource-poor settings where one or more physicians review the VA data to assign a probable cause of death [3, 29, 30].

Data analysis was conducted using STATA and ArcGIS software. Maternal mortality rate in this study is defined as the number of maternal deaths in a given period (2006–2010) per 1 000 women of reproductive age during the same time-period. It is calculated by dividing the number of maternal deaths by the total person-years lived by the women and expressed per 1000 person-years. Proportional maternal mortality by cause and frequency were generated. Cause-specific maternal mortality rates for each calendar year were calculated and stratified by age, using person-years analysis. The associations between maternal death and risk factors were assessed using survival analysis with non-parametric Cox proportional hazards regression to predict or model maternal mortality from independent variables in univariable and multivariable models, taking into account possible confounders and interaction factors. The assumption of proportional hazards was tested using the stphtest command in STATA that is based on the Schoenfeld residuals. Only variables with p values of 5% or less in the univariable analysis model were included in the multivariable analysis.

As the study used longitudinal data, use of event history analysis, which is due to continuous changes in the risk of pregnancy and maternal mortality as women grow older, was essential. Therefore, a person-time contribution for every woman, as part of the denominator, was critical. Hazard ratios (HR) with 95% confidence intervals were calculated. A final multivariable model was fitted, and the maternal mortality trend over the five year period was determined.

ArcGIS 9.1 software was used to generate interpolated maps covering the same geographical extents as the study area, based on the number of deaths in women. Based on the assumption that geographical features near each other are likely to be more related than features that are distant [31, 32], mortality distribution was determined, using the inverse distance weighted (IDW) method of interpolation in ArcGIS. This assigns values to locations using a deterministic interpolation model to produce a surface pattern based on how far locations are to sentinel locations where measurements have been taken. In this way, the interpolation surface was used to make predictions from the households where the deaths occurred, for all locations in a raster dataset, representing the study area [33]. The interpolation maps were then visually inspected to identify similarities in the location of distinctive hotspots (areas with highest mortality rates), indicating the spatial pattern of deaths of women and maternal deaths in the study area relative to the distances from the homes of women to the nearest health facilities.

## Results

### Socio-economic and demographic characteristics

Table 1 presents the background characteristics of the research participants.

Most of the women (72.7%) were 20–39 years old; most were married (94.7%), and most earned their livelihoods through subsistence farming (82.5%). The highest proportions were in the two lowest SES quintiles (29.5% and 31.0%, respectively). Many women (35.9%) had no formal education; 53.8% had only primary level education. Most (41.2%) had had five or more pregnancies.

**Table 1:** Socio-demographic characteristics of women aged 15 to 49 years in IHDSS, 2006–2010.

Maternal mortality Risk factors	Number (n)*	Proportion (%)
**Maternal Age (years)**		
< 20	4218	11.5
20–29	16042	43.6
30–39	10715	29.1
40–49	5817	15.8
**Marital status**		
Single	567	1.5
Married	34821	94.7
Divorced/Separated	1179	3.2
Widowed	224	0.6
**Occupation**		
Business	98	1.3
Employed	167	2.3
Farming	6136	82.5
Fishing	104	1.4
Others	361	4.9
Trading	571	7.7
**Education**		
No Education	8381	35.9
Primary	12549	53.8
Secondary and above	2399	10.3
**Parity**		
Parity 1	2962	21.8
Parity 2–4	5044	37.0
Parity 5+	5610	41.2
**Number of antenatal clinic visit**		
4 and above	1919	14.1
0, 1	9490	69.7
2–3	2214	16.3
**Who assisted in delivery**		
Health worker	6895	66.0
Non-health worker	3548	34.0
**Delivery place**		
Health facility	6897	62.2
Outside health facility	4184	37.8
**Distance of woman's home to health facility**		
Near (< 5Km)	819	80.7
Far (> = 5Km)	196	19.3
**Total person years**	**97999.54**	

Note

*For each risk factor, number of women varies due to missing values

Most of the women (69.1%) reported having visited the antenatal clinic only once during their last pregnancy, or not at all. Sixty-two percent were assisted by a health worker (doctor or nurse) during their last delivery; 62.2% delivered in a health facility (indicating that they had access to health facilities). Most of the women (80.7%) lived less than 5 km from the nearest health facility as shown in Table 1.

### Maternal mortality rate, spatial patterns and trends

The women in the study contributed 97 999.54 person-years of observation. There were 77 maternal deaths (0.2%) amongst the 36 792 women; 2 271(6.2%) died from other causes. There was a downward trend in the proportion of maternal deaths across the study period. The largest proportion occurred in 2007 (27.3%), 26.0% occurred in 2006, 16.9% in 2009, 15.6% in 2010, and 14.3% in 2008. The maternal mortality rate was estimated as 0.79 deaths per 1000 person-years of observation (95% CI 0.63–0.98) over the five year period.

**Fig 2:**
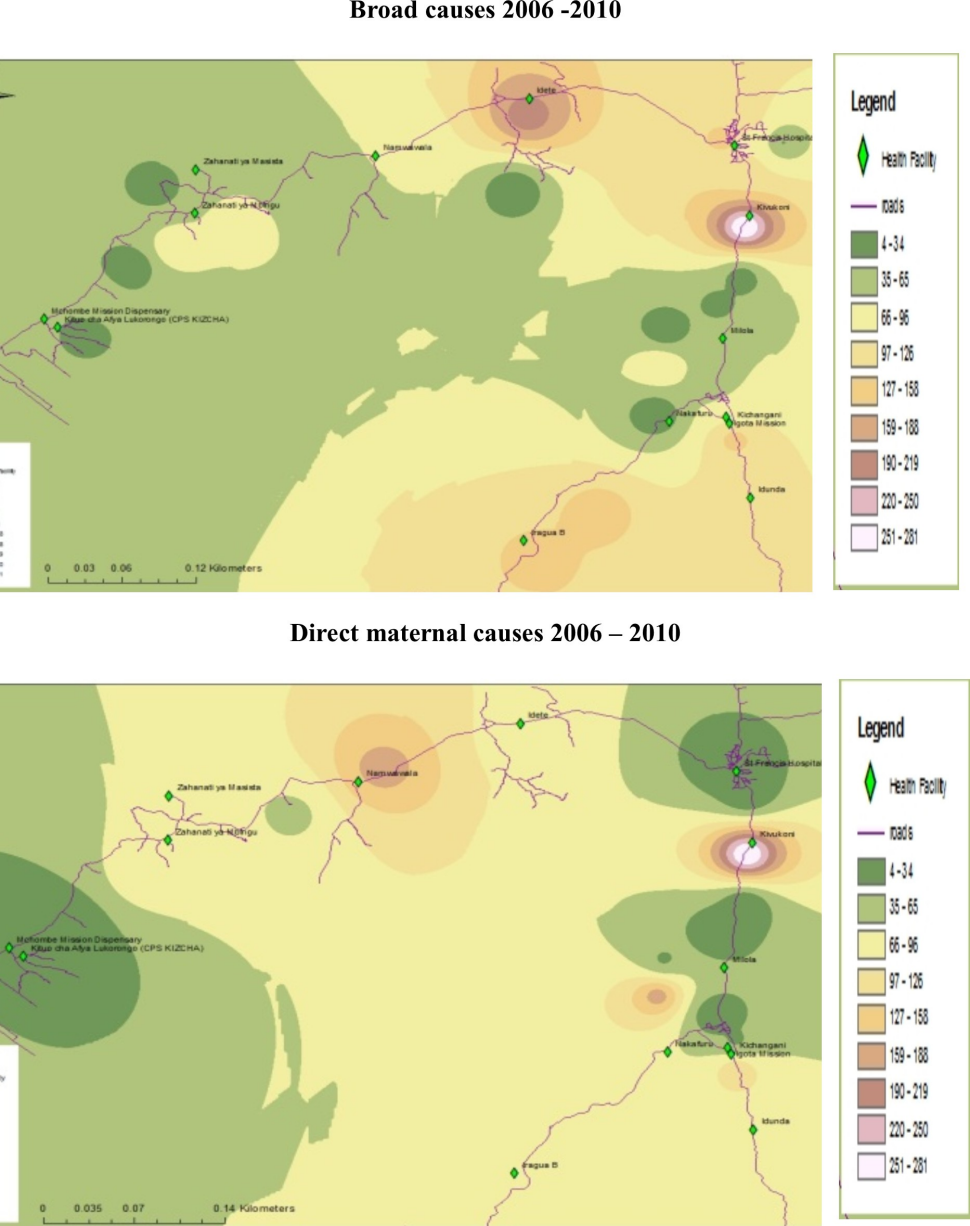
Mortality patterns from 2006–2010 in IHDSS.

**Fig 3:**
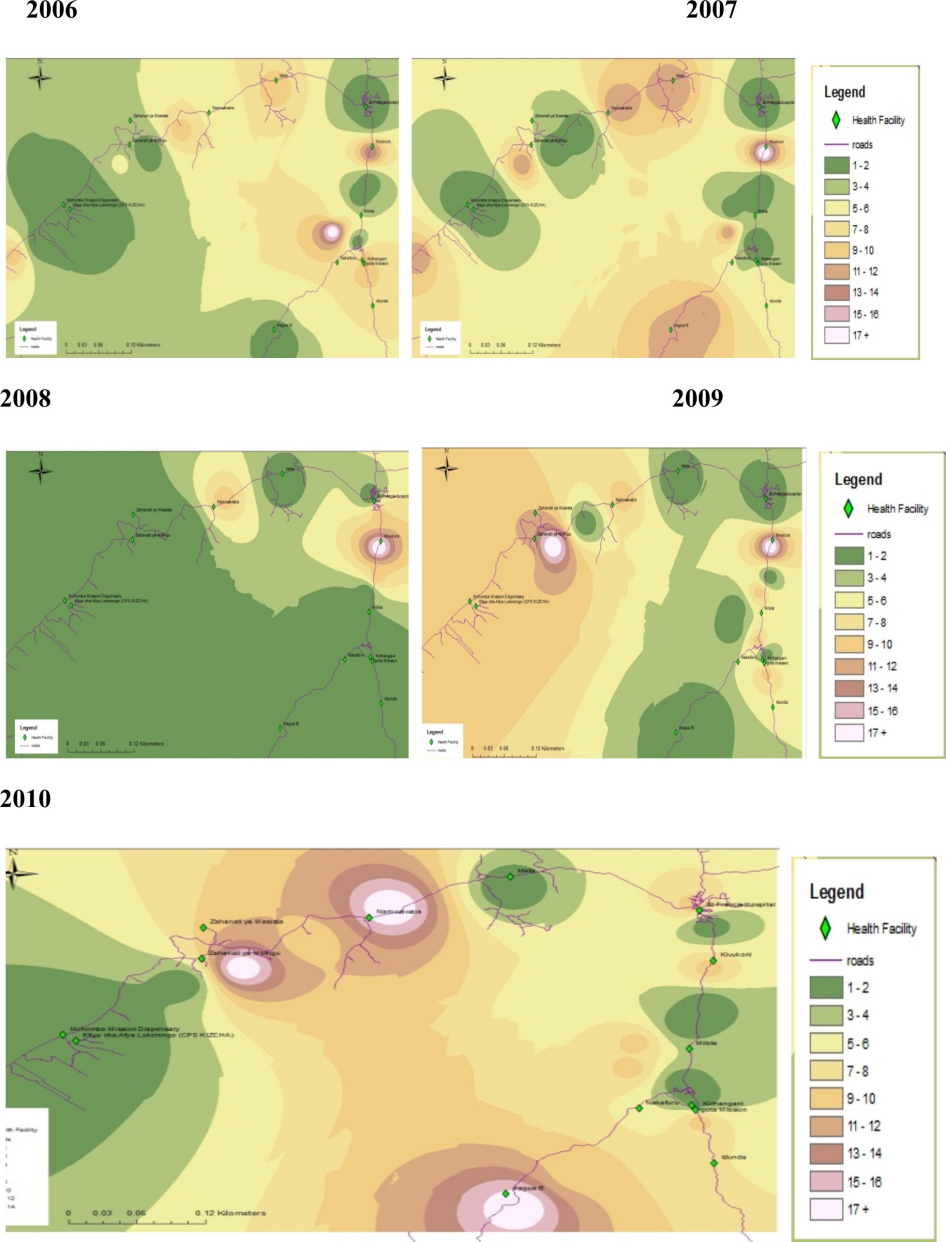
Yearly maternal mortality patterns in IHDSS, 2006–2010.

There were marked geographical differences in both the broad causes of mortality and direct maternal causes with regard to spatial patterns, even within a relatively small area (Figs 2 and 3). Most of the deaths occurred in close proximity to Kivukoni, Namwawala, Mbingu and Idete health facilities.

**Fig 4:**
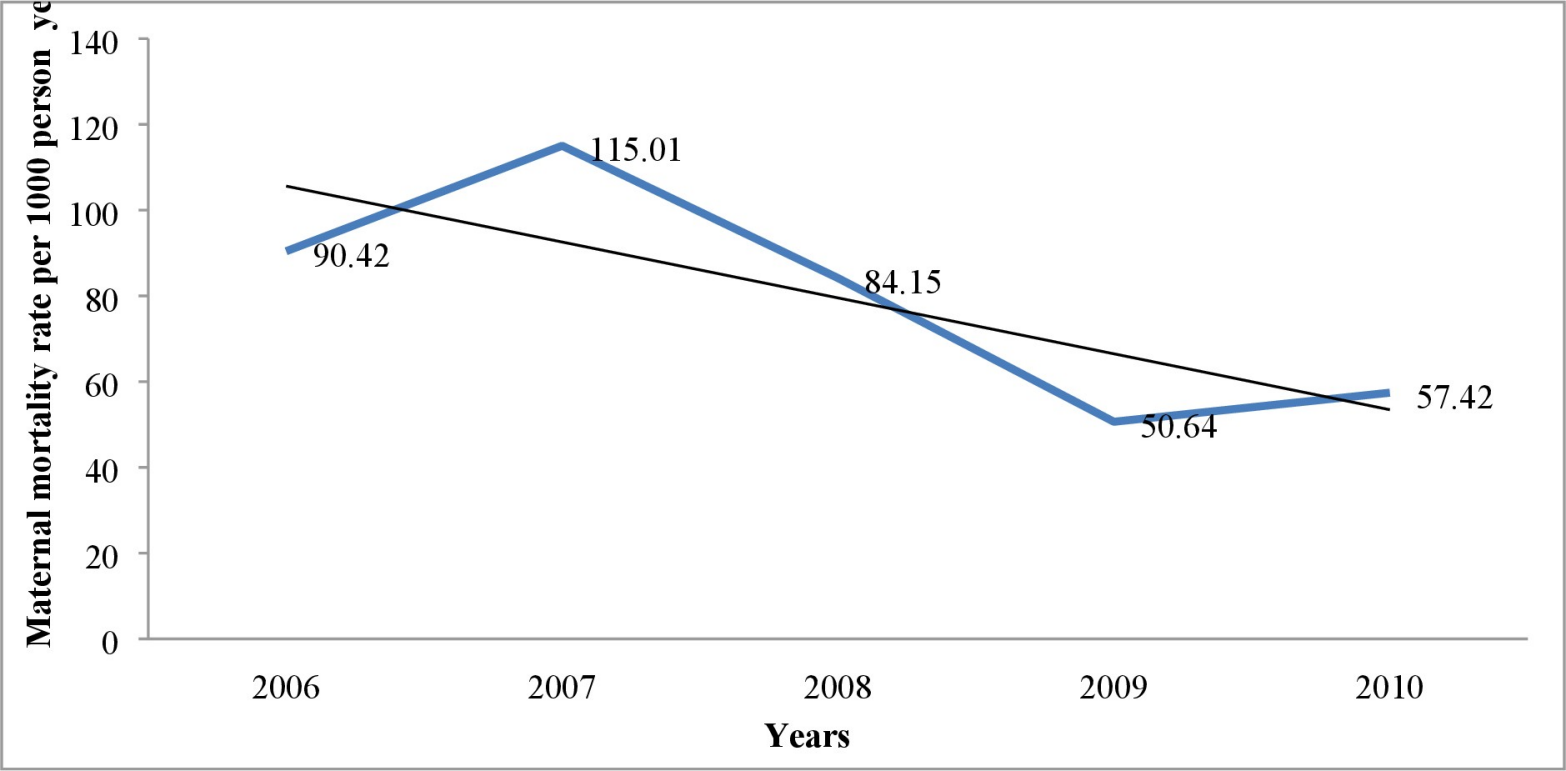
Maternal mortality trend from 2006–2010 in IHDSS.

Fig 4 reveals that the maternal mortality rate declined in the IHDSS over the five year period, from 90.42 in 2006 to 57.42 in 2010, with a gradient of -13.04 on a linear scale.

### Causes of death in women

Malaria accounted for 13% of the total mortality burden (2 348 deaths), followed by cardiovascular diseases which contributed 10% of the total deaths. HIV/AIDS and pneumonia each accounted for 8% of deaths while anaemia contributed 6%. External and pregnancy-related deaths contributed 3% each. Of the 77 maternal causes of death, eclampsia and haemorrhage accounted for 23% and 22% respectively, followed by abortion complications (10%), and obstructed labour (7%). Other specified and unspecified direct causes contributed 37%; puerperal sepsis contributed only 1%.

In 2006, abortion and haemorrhage were the main causes of mortality, followed by eclampsia and obstructed labour. Elampsia was the main cause in 2007, followed by unspecified maternal causes and other specified causes. In 2008, other specified causes and unspecified causes were the major contributers to mortality. In 2009, haemorrhage was the major cause while, in 2010, unspecified direct causes and eclampsia were the two main causes.

In the study period, 46 of the women died from non-obstetric causes. Anaemia contributed the largest proportion (22%), followed by HIV/AIDS (15%), cadiovascular disease (13%), and malaria (7%). Of the 72 external causes of death, other specified unintentional injury contributed 42%, road traffic accidents 19%, homicide 14%, assault 12%, accidental poisoning 7%, and suicide and falls 3% each.

### Risk factors of maternal mortality

The results from the univariable Cox regression model (Table 2) show that maternal age, socio economic status, marital status, birth attendant, place of delivery and parity were statistically significant risk factors for maternal mortality in the IHDSS from 2006 to 2010. Women aged 20 to 29 years were 43% less likely to experience maternal mortality compared to those younger than 20 years (HR = 0.57, 95% CI: 0.16–1.99). Those aged 30 to 39 years were 3% more likely to experience maternal death (HR = 1.03, 95% CI:0.30–3.49), while older women (40–49 years) were 80% less likely to die from maternal causes of death (HR = 0.20, 95% CI: 0.04–1.00).

**Table 2:** Univariable hazard ratios of maternal mortality risk factors in IHDSS, 2006–2010 (unadjusted).

Maternal mortality risk factors	Hazard Ratio (HR)	P Value	95% CI
**Maternal Age (Years)**			
< 20 (Reference)	1.00		
20–29	0.57	0.377	(0.16–1.99)
30–39	1.03	0.968	(0.30–3.49)
40–49	0.20	0.050 †	(0.04–1.00)
**Socio economic status**			
Poorest (Reference)	1.00		
poorer	0.16	0.000 †	(0.06–0.42)
poor	0.29	0.005 †	(0.12–0.69)
less poor	0.37	0.169	(0.09–1.53)
least poor	0.28	0.017 †	(0.10–0.80)
**Education**			
No Education (Reference)	1.00		
Primary	0.77	0.324	(0.45–1.29)
Secondary and above	0.53	0.389	(0.13–2.24)
**Occupation**			
Business (Reference)	1.00		
Farming	0.25	0.174	(0.03–1.86)
Others	5.71	0.087	(0.70–42.05)
Trading	1.55	0.674	(0.20–11.71)
**Marital Status**			
Single (Reference)	1.00		
Married	0.05	0.000 †	(0.01–0.25)
Divorced/Separated	8.64	0.003 †	(2.08–35.98)
Widowed	5.60	0.040 †	(1.09–28.86)
**Number of antenatal clinic visit**			
4 and above (Reference)	1.00		
0 to 1	1.12	0.848	(0.35–3.64)
2 to 3	0.91	0.897	(0.23–3.65)
**Assistant at delivery**			
Health worker (Reference)	1.00		
Non health worker	1.84	0.012 †	(1.14–2.98)
**Delivery place**			
Health facility (Reference)	1.00		
Outside health facility	1.68	0.032 †	(1.05–2.69)
**Parity**			
Parity 1 (Reference)	1.00		
Parity 2–4	2.97	0.043 †	(1.04–8.51)
Parity 5+	5.29	0.002 †	(1.84–15.25)
**Distance from woman's home to HF**			
Near (< 5Km) (Reference)	1.00		
Far (> = 5Km)	0.98	0.929	(0.57–1.67)

Note

† Statistically significant, HF = Health Facility

Higher SES was protective against maternal mortality. Women who belonged to the poorer SES had an 84% reduction in maternal mortality compared with those in the poorest category (HR = 0.16, 95% CI: 0.06–0.42); there was a 71% reduction in the poor SES category (HR = 0.29, 95% CI: 0.12–0.69), a 63% reduction in the less poor SES category (HR = 0.37, 95% CI: 0.09–1.53), and a 72% reduction in the least poor category (HR = 0.28, 95% CI: 0.10–0.80).

Marital status was also associated with maternal mortality. Married women appeared to have a much lower risk of maternal mortality compared to single women (HR = 0.05, 95% CI: 0.01–0.25), while those who were divorced/separated or widowed had an increased risk (HR = 8.64, 95% CI: 2.08–35.98; and HR = 5.60, 95% CI: 1.09–28.86, respectively).

Women who were assisted in their deliveries by non-health workers were 84% more likely to experience maternal mortality than those who were assisted by a health worker (HR = 1.84, 95% CI: 1.14–2.98). Women who delivered outside a health facility were also at a 68% higher risk of maternal mortality compared to those who delivered in a health facility (HR = 1.68, 95% CI: 1.05–2.69). As parity increased, so did the risk of maternal mortality.

The multivariable Cox proportional hazard model (Table 3) showed that maternal age, marital status and socioeconomic status were statistically significantly associated with maternal mortality. Maternal age was a significant risk factor in that women younger than 20 years were at the highest risk of maternal mortality. There was no trend in risk in the other age groups.

**Table 3:** Multivariable hazard ratios of maternal mortality risk factors in IHDSS, 2006–2010.

Maternal mortality risk factors	Hazard Ratio (HR)	P–Value	95% CI
**Maternal Age (Years)**			
< 20	1.00		
20–29	0.18	0.018 †	(0.05–0.74)
30–39	0.22	0.038 †	(0.05–0.92)
40–49	0.21	0.080	(0.04–1.20)
**Marital Status**			
Single	1.00		
Married	0.06	0.010 †	(0.01–0.51)
Divorced/Separated	7.15	0.055	(0.96–53.41)
Widowed	9.13	0.048 †	(1.02–81.94)
**Parity**			
Preg1	1.00		
Preg2-4	1.94	0.433	(0.37–10.12)
Preg5+	2.25	0.328	(0.44–11.46)
**Who assisted in the delivery**			
Health worker	1.00		
Non health worker	5.83	0.104	(0.70–48.90)
**Delivery place**			
Health facility	1.00		
Outside health facility	0.28	0.241	(0.04–2.33)
**Socio economic status**			
poorest	1.00		
poorer	0.30	0.018 †	(0.11–0.81)
poor	0.48	0.115	(0.20–1.19)
less poor	0.48	0.328	(0.11–2.10)
least poor	0.25	0.065	(0.06–1.09)

Note

†: Statistically significant

Married women had the lowest risk of maternal mortality. Women with the lowest SES had the highest risk of maternal mortality. There was no decreasing trend in risk with increase in SES.

Women who were assisted with their deliveries by non-health workers were 5.83 times more likely to experience maternal death (HR = 5.83%, 95% CI: 0.70–48.90) compared to those who were assisted by a health worker, although this was not statistically significant.

The proportional hazard assumption was investigated in the model and it was upheld with a global test of p = 0.9175.

## Discussion

Maternal mortality was not evenly distributed across the study area in the five year study period. There were areas in the DSA with higher numbers of maternal deaths than others, supporting results from a similar infant mortality study in South Africa [18]. Since understanding spatial patterns of a health-related problem forms the most basic tenet of public health [34, 35], this analysis has provided evidence for the need to target intervention programmes to high risk areas where health events are most likely to occur, since population-wide interventions may be too costly to implement. Studies have shown that community-level interventions bring about reduction in maternal mortality [36].

Distances to the nearest primary health care facility and the number of antenatal clinic visits attended were not significant risk factors for maternal mortality. The understanding of other factors that may obstruct access to health care utilization, such as quality of care, level of available care, cost, and social and behavioral factors, is important, as suggested in other studies [18, 37].

There was a declining trend in maternal mortality over the five year period, in line with the declining global trend and the trend in Tanzania in general [38, 39]. This trend could be attributed to the various interventions that have been put in place in the last few years at both the national and the district level in Tanzania, such as improved and wide availability of Emergency Obstetric Care (EMOC) [40] and a higher proportion of facility-based deliveries [41]. Although it was difficult to compare the maternal mortality findings in this study with other studies, due to different scales of measurement used (rates and ratios), declining patterns have also been reported at the national level [4].

The major direct cause of maternal mortality in IHDSS were eclampsia and haemorrhage, similar to reports in other studies [39, 42]. In our study abortion, related complication is the third direct course of maternal death. This finding is consisted with other studies in Tanzania where unsafe abortion represents one of the leading causes of maternal deaths in Tanzania [43, 44].

The main indirect causes of maternal death were anemia, HIV, cardiovascular diseases, and malaria. This supports the report that HIV and cardiovascular diseases are among the leading causes of mortality in Sub-Saharan Africa [45]. HIV has been shown in others studies in Tanzania and Sub-Saharan Africa to be among the leading causes of mortality [46–48]. Our study also shows the changing pattern of causes of mortality in Africa where deaths due to malaria are decreasing and HIV and cardiovascular disease are becoming the predominant causes [46]. This is a challenge to the attainment of SDG 3.1 since the prevalence of HIV is still high.

Road traffic accidents, homicides and assaults were the three leading external causes of death of women in this study. This is in line with the global pattern of mortality due to road traffic accidents and injuries [49–51]. Injuries, suicides, homicides and other specified unintentional traumatic injuries have been an increasing source of maternal mortality in recent times [52], consistent with our study. This was primarily due to the inclusion of late maternal deaths into the maternal mortality estimates. It is important to include violent deaths in pregnancy in official statistics on maternal mortality to guide appropriate care and intervention programs. The present global priorities for adolescent health policy, which focus on HIV/AIDS and maternal mortality, are an important but insufficient response to prevent mortality in an age-group in which more than 40% of deaths are due to intentional or unintentional injuries [51].

We found that maternal age, marital status, and SES are significantly associated with maternal mortality in IHDSS. This is consistent with findings from other studies [51, 53, 54] where the risk of maternal mortality was highest for adolescent girls and where complications in pregnancy and childbirth were the leading cause of death in the age group in most developing countries [51]. In our study, the risk of maternal death was lower in women who were older than 20 years of age. A study conducted in Ghana, Kenya and Malawi [55] suggested that due to social ramification of teenage pregnancy which includes expulsion from school and stigma, adolescents are at risk of delaying pregnancy disclosure and to initiate ANC attendance in the first trimester of pregnancy which exposes them to the risk of pregnancy related complication. This may increase the risk of maternal death among adolescent. The increase risk of maternal death among the adolescent in this study could also be due to lack adequate ANC visit among the study participants as shown by at studies from Ababa and Nigeria [56, 57].

Urassa *et al*. reported that single and divorced women were at risk of maternal death in Dar es Salaam [58]. We found a similar result, where married women were at a 94% lower risk of maternal death compared to women who were single. Similar to our findings, other studies found increased risk of maternal death with increasing parity [58, 59]. Loudon *et al*. [60] also, showed that for all ages, there was a higher risk of maternal death in first births than in the second or third. From the fourth birth onwards, increasing parity led to higher maternal mortality, regardless of maternal age. Likewise, for all parities, maternal mortality increased with age.

Studies have shown that household social class measure is a better predictor of reproductive outcomes than individual social class standing [61]. Urassa *et al*. [58] showed that factors reflecting living standards, such as type of housing, access to tap-water, electricity, availability of a toilet, and the living standard as estimated by the interviewer were all statistically significant for the risk of maternal death. The findings are consistent with our results, where factors reflecting living standards of women’s household were used as a proxy for maternal SES, and women with higher SES were at lower risk of maternal mortality compared with those of very poor SES.

While women's education appeared to not be a risk factor for maternal death, previous studies have shown conflicting results. Some reported similar results to ours [59, 62, 63] but others found that maternal education does exert an effect on maternal mortality [58, 64, 65].

Some studies have reported that home delivery and inadequately trained birth attendants are risk factors for maternal death [66, 67]. Results of our study point in the same direction but without a statistical significance, women who were assisted by non-health workers during delivery were 5.83 times more likely to experience maternal death compared to those who were assisted by health workers. Again, while distance from women’s homes to the nearest health facility was not a risk factor in our study, Wagle *et al*. [66] found that it was a risk factor in Nepal.

## Limitation and strength

Other maternal mortality risk factors have been reported in the literature but this was a secondary analysis of data and we did not have information on other potential predictors.

In considering the findings of this study, it is important to bear several issues in mind. The large population under surveillance in the IHDSS and the rigorous demographic surveillance system which continuously captures vital population statistics (births, deaths and migration) longitudinally provides a platform for a reliable person-time of exposure which enables calculation of accurate maternal mortality rates, free from the influence of stillbirth prevalence and induced abortion that are present in maternal mortality ratio calculations [68]. The use of a population-based sample limits the issue of selection bias that would otherwise be introduced by hospital-based studies and the results obtained are consistent and comparable with scientific findings in other settings. Hence, the authenticity of the results was not compromised.

## Conclusion and recommendation

Although there appears to be a declining trend of maternal mortality in southern rural Tanzania, there are marked geographical differences in maternal mortality, with variations across a relatively small geographical area. The high levels of maternal mortality in some instances occurred in homes in close proximity to health facilities, suggesting a need to strengthen the capacity of sub-district health facilities for emergency obstetric care, to improve quality of care and the level of available care, and to assess cost, social and behavioral barriers that might obstruct access to health care services.

For Tanzania to meet the SDG 3.1 target of reducing MMR to below 70 maternal deaths per 100,000 live births by the year 2030, there is the need for commitment and effective implementation of the “National Road Map Strategic Plan to Improve Reproductive, Maternal, Newborn, Child & Adolescent Health in Tanzania (2016–2020)”[69] and the “Health Sector Strategic Plan July 2015 –June 2020”[70] is crucial. Reaching teenagers with friendly reproductive health services as explicitly stated in those national strategies could contribute to reducing the maternal mortality through delaying first birth beyond the risk age (teenage). The two leading causes of maternal mortality were eclampsia and haemorrhage. Proven interventions to control these causes need to be available at health facilities.

The observed spatial differentials in the maternal deaths across a relatively small geographical area call for a comprehensive mixed-methods study to understand the causes.

## Supporting information

S1 File Supporting data file. (DTA)
